# A Peridynamics-Based Micromechanical Modeling Approach for Random Heterogeneous Structural Materials

**DOI:** 10.3390/ma13061298

**Published:** 2020-03-13

**Authors:** Sumeru Nayak, R Ravinder, N M Anoop Krishnan, Sumanta Das

**Affiliations:** 1 Civil and Environmental Engineering, University of Rhode Island, Kingston, RI 02881, USA; sumeru@uri.edu; 2 Department of Civil Engineering, Indian Institute of Technology Delhi, New Delhi, India; cez177518@iitd.ac.in

**Keywords:** state-based peridynamics, micromechanical modeling, critical stretch, random heterogeneous structural materials, particulate reinforced cementitious composites

## Abstract

This paper presents a peridynamics-based micromechanical analysis framework that can efficiently handle material failure for random heterogeneous structural materials. In contrast to conventional continuum-based approaches, this method can handle discontinuities such as fracture without requiring supplemental mathematical relations. The framework presented here generates representative unit cells based on microstructural information on the material and assigns distinct material behavior to the constituent phases in the random heterogenous microstructures. The framework incorporates spontaneous failure initiation/propagation based on the critical stretch criterion in peridynamics and predicts effective constitutive response of the material. The current framework is applied to a metallic particulate-reinforced cementitious composite. The simulated mechanical responses show excellent match with experimental observations signifying efficacy of the peridynamics-based micromechanical framework for heterogenous composites. Thus, the multiscale peridynamics-based framework can efficiently facilitate microstructure guided material design for a large class of inclusion-modified random heterogenous materials.

## 1. Introduction

The classical theory of solid mechanics, because of its reliance on partial differential equations, is inherently limited when applied to failure of materials [1,2,3]. The non-existence of the spatial derivatives at the crack tips introduces singularity, which is alleviated with supplemental relations for stable numerical modeling. This necessitates a reformulation of the fundamental equations of continuum mechanics for universal application regardless of discontinuities arising from deformations. To address this, an alternative approach called peridynamics has been proposed, which uses integral equations and maintains the integrity of the mathematical structure in the event of a discontinuity [4,5,6].

In peridynamics, any failure is treated as a natural outcome of the deformation arising out of the equations of motion and the constitutive model [2]. This eliminates the need for supplemental kinetic relations which would otherwise be necessary in fracture mechanics to define crack initiation and propagation [7,8]. Thus, peridynamics has been gaining traction owing to its ability to handle multiple scales with long-range forces that can be efficiently integrated in a constitutive model. In contrast to contact forces in classical methods, peridynamics considers the forces between particles beyond the immediate neighbor, as though they act across a finite distance. This non-locality contributes to the robustness of peridynamics in handling multiple interactive scales [9,10].

Broadly, peridynamics can be classified into bond-based and state-based models. Bond-based peridynamics was initially developed [5,11] and was restricted to only central force loading and a Poisson’s ratio of ¼ [1,12]. A more robust formulation was later introduced in the form of state-based peridynamics [1,13] that can capture volume changes thereby overcoming the limitations posed by the bond-based theory. In the state-based peridynamics, the forces between the peridynamic nodes are not only governed by those particles but also by the surrounding bonds. Additionally, the introduction of force and deformation states in the state-based model allows easier correlation with classical continuum mechanics and easy import of classical constitutive relationships into the framework, thereby reconciling the peridynamic theory with classical mechanics [14]. The wide applicability of peridynamic formulation is exemplified by its usage for modeling fiber reinforced composites [15,16,17], polycrystals [15,18,19] and macro-scale concrete structures [10,20].

While previous studies mostly correspond to macro-scale analysis of such materials, the current study implements a multiscale state-based peridynamic framework towards effective property computation in random heterogeneous structural materials. Towards that end, a numerical simulation framework is proposed in the current study involving multi-phase random microstructures which are assigned peridynamic material models and solved at different interactive length scales. To predict the constitutive behavior and fracture response of highly heterogenous structural materials, the challenge lies in establishing techniques that can handle more than the phase volume fractions. Analytical homogenization techniques [21,22,23] resulting in closed form solutions are often incapable of post-peak response prediction and are rendered ineffective while handling distinct phases with significant stiffness contrasts [24,25]. Lately, computational techniques towards enhanced accuracy have been implemented [25,26,27,28,29]. Such techniques include Lattice approach demonstrating meso-scale simulations [30,31,32] with discrete elements and Finite element method (FEM) involving additional kinetic relations for failure initiation and propagation [33,34]. The peridynamic formulations eliminate the need for such explicit relations. In addition, in case of modelling of interfacial characteristics in heterogeneous systems, FEM-based cohesive zone models (CZM) are often plagued by mathematical and physical limitations apart from constraints on cohesive laws and loss of accuracy at crack tips in extended finite element method (XFEM) that explicitly define the nature of crack initiation and propagation [35,36,37]. The implementation of peridynamics not only does away with such ad hoc postulations used in classical approach but also enables a mesh-free discretization thereby eliminating the need for computationally expensive meshing algorithms.

This study applies the proposed peridynamics-based multiscale framework towards prediction of an effective mechanical response of a metallic particulate incorporated cementitious composite. Such heterogenous systems have been established by experimental observations [38] and integrated in FE-based multiscale simulations [39] to elucidate the enhanced fracture performance. The peridynamic approach adopted here enables a mesh-free discretization and implements a critical stretch-based failure criterion, in contrast to the explicit damage initiation and propagation laws adopted in the erstwhile FE study. The interfaces in the three-phase microstructures are handled as distinct material domains by the current approach in contrast to FE simulations whereby cohesive elements with predefined traction-separation behavior are implemented. The peridynamic formulations enable autonomous failure propagation without the need for defining damage laws, thus facilitating the computation of constitutive relationships of particulate incorporated cementitious systems. The predicted responses are compared with macro-scale experimental results to assess the effectiveness of the proposed framework. Thus, the proposed peridynamics-based framework is expected to enable efficient design of a sizable class of inclusion-reinforced random heterogeneous cement-based composites for various applications which is the ultimate objective of this research work.

## 2. Peridynamics-Based Numerical Simulation Framework

**Figure 1:**
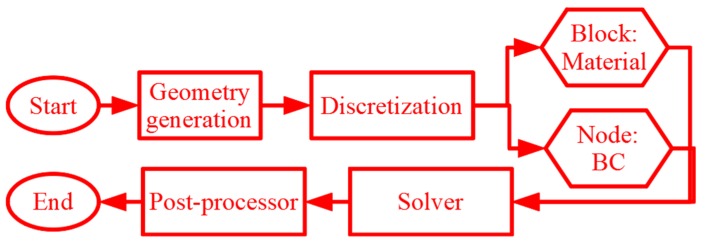
A schematic framework for peridynamics-based micromechanical modeling.

This section elaborates the peridynamics-based numerical simulation framework. The framework involves generation of discretized unit cells accurately representing the multi-phase material microstructure followed by a peridynamics-based micromechanical analysis. The material definitions and damage models are implemented in the discretized domain and an explicit solver in the open source code Peridigm [13] is used to obtain the engineering stresses and strains. A post-processing module enables visualization of the deformed unit cells and the computation of effective constitutive response. The surface effects observed in such simulations are eliminated by the implementation of influence functions in the force state that can effectively incorporate the presence of interfaces and boundaries. A schematic for the numerical simulation framework is shown in Figure 1.

### 2.1. Generation of Representative Unit Cells

The representative unit cell generation is carried out using Lubachevsky–Stillinger algorithm [29,40,41]. The algorithm implements random packing of rigid particles which are not allowed to overlap owing to hard contact model. Random positions and velocities are assigned to the particles with zero initial radius. At time *t* = 0, the initial velocities of the infinitesimal points have randomly distributed components between −1 and +1. As the points begin to grow into particles, their sizes at any instant of time are governed by a continuous nondecreasing function of the growth rate (*g_i_*) for every *i^th^* particle. The growth of the particles results in collisions, which in turn determines the subsequent velocities of the particles. In an iterative framework, the frequency of such collisions increases with increase in the size of the particles. The formulations of the iterative framework that terminates at a target volume fraction, as presented in [39], places the particles at various positions in the bounding box, which thereafter collide and grow so as to achieve the target volume fraction [42]. The iterative algorithm is terminated as the inclusions occupy the target volume fraction. As the terminating criterion is met, the positions and velocities of the particles are frozen. Thus, the final states of the particles in the representative geometry are obtained. The representative unit cells are periodic in nature [43,44,45,46,47] implying material continuity at the boundaries. The information pertaining to the final particle radii and their locations along with their orientations are passed as input parameters to the discretization module. The formulation is implemented in a python script that outputs the final states of the particles in the generated unit cell.

### 2.2. Discretization Module and Boundary Conditions

The microstructural information obtained from the unit cell generation framework is passed on to the discretization module. The numerical implementation of the discretization procedure commences with the definition of a bounding box with dimensions equal to that of the unit cell. Thereafter, the module discretizes the unit cell domain into nodes, each with a known volume that serves as the reference configuration for the ensuing Peridynamics framework. This discretization method is meshfree [2] as there are no elements or geometrical connections between the nodes. Such a discretization method enables efficient computation of forces at a node in the Peridynamics-based framework where the influence of only the surrounding nodes lying within the horizon are considered. The nodes in the discretized unit cell domain are assigned different sets of materials IDs so as to represent the shapes and orientations of the different component phases in the generated representative unit cell (Section 2.1) effectively. This facilitates application of relevant material and damage models to various phases in a random heterogenous microstructure in the Peridynamics code. The meshfree discretization is performed using a python script. The generated meshfree representative unit cell is used in the Peridynamics code.

The boundary conditions of the unit cell are imposed on a volume of boundary layers equaling the horizon. To implement periodic boundary conditions, a necessary condition is to ensure the continuities of displacement and traction. Along the boundary of the material region, the displacement boundary conditions are implemented on the boundary layer (with a depth equaling horizon as mentioned earlier). In the boundary layer, constraints are assigned to the material points in the region. The displacements thus assigned to the layer are linear approximations of the boundary displacement (as applied on the unit cell). Traction conditions can be similarly enforced, which in the present scenario is zero and can be implemented naturally. To ensure the continuity of both displacement and traction at surfaces defining the unit cell, the family of material points are generated in a periodic array. Once the boundary is encountered at a corner or surface, a cut-off procedure is adopted and the surplus material points are impressed on the opposing face or corner. The periodic conditions are thereafter adopted by constraining the mapped points on opposite faces or corners. The uniaxial response prediction is implemented by imposing a relative displacement between two such points (in a plane perpendicular to the direction of loading) lying on opposite faces.

### 2.3. Peridynamics-Based Micromechanical Modeling

**Figure 2:**
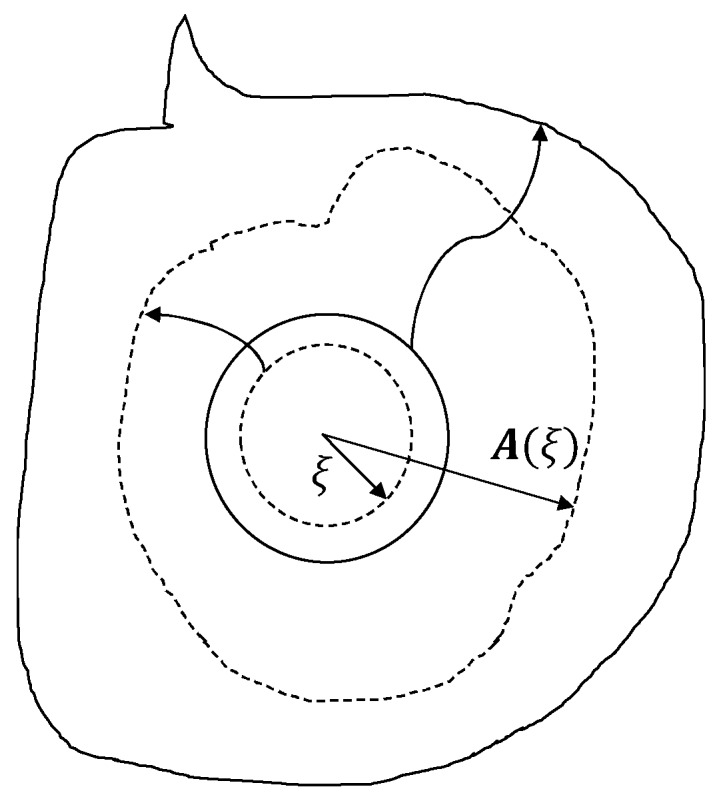
A vector state **A** mapping a sphere into a complex surface.

The state-based generalization of the peridynamics is used in this paper. In this state-based framework, the response of a material at a point depends collectively on the deformation of all bonds connected to the point which is ensured by defining mathematical objects called deformation state and force state. While the deformation state contains the deformed configurations of the bonds, the force state contains the forces in all these bonds. The constitutive material model relates the deformation and force states. The kinematics of the peridynamic method are captured in a discretized domain (see Figure 2). Each material point *x* in the discretized domain has a finite volume. It interacts with other points *x*’ which are located within a specific region *H_x_* (family of *x*). This region is considered to be a sphere centered at point *x* with a radius *δ*, referred to as the horizon. The position vector state *X*, also referred to as the bond between the particles [1,48], captures the relative positions of the interacting particles in the undeformed configuration. The position vector state corresponding to the bond *x*’ − *x* is denoted by *X*<*x*’ − *x*>. Once the deformation sets in, the relative displacements between the two points is defined by *η* (refer to Equation (1)). Thereafter the deformation vector state *Y*<*x*’ − *x*> is defined as the sum of *X*<*x*’ − *x*> and ***η*** (see Equation (2)).

(1) $$\eta =u\left(x^{\prime },t\right)-u\left(x,t\right)$$

(2) $$Y\langle x^{\prime }-x\rangle =X\langle x^{\prime }-x\rangle +\eta$$

where *u* is the displacement vector field. The peridynamics-based simulations are governed by the equation of motion, derived from the conservation of linear momentum [1,7], as shown in Equation (3) [2].

(3) $$\rho \left(x\right)\ddot{u}\left(x,t\right)=\int_{H_{x}}\{T\left[x,t\right]\langle x^{\prime }-x\rangle -T\left[x^{\prime },t\right]\langle x-x^{’}\rangle \}dV_{x^{\prime }}+b\left(x,t\right)$$

where *ρ* is the local density, ü(x, t) is the acceleration of point x at time t, b is the external body force density, dV**_x’_** is an infinitesimal volume around x’ and T is the force vector state that describes interaction between points. The force state T[x, t] at a point x at time *t* is a function that associates a force density to the bond *x*’ − *x* acting at *x.* The force density arises due to the internal forces generated by deformation of family of *x* (points within the horizon of *x* in the reference configuration) relative to *x*. Since T[x, t] depends only on the deformation of the family of *x*, it assumes a zero value for any bond beyond the horizon. The forthcoming subsections elaborate the peridynamic formulations in the context of material properties.

#### 2.3.1. Material Model

For an ordinary state-based peridynamic formulation [1], a material is ordinary if the force state *T* for any deformation has the same direction as that of the deformation state *Y* as shown in Equation (4).

(4) $$T\langle x^{\prime }-x\rangle =C\frac{Y\langle x^{\prime }-x\rangle }{\mathrm{||}Y\langle x^{\prime }-x\rangle \mathrm{||}}$$

where *C* is a scalar force state and $\frac{Yx^{\prime }-x}{\mathrm{||}Yx^{\prime }-x\mathrm{||}}$ is the unit vector that points from the deformed position of *x* towards the deformed position of *x*’. For ordinary state-based model, Equation (4) is valid for ‖*Y*<*x*’ − *x*>‖ ≠ 0. Otherwise, a zero value is considered for *T*<*x*’ − *x*>. Thus, in an ordinary material the direction of *T* matches with the direction of *Y* for any bond where *C* ≠ 0 (undamaged configuration). For an elastic material, a differentiable scalar valued function *W* exists as shown in Equation (5).

(5) $$T=\hat{T}\left(Y\right)=\nabla W\left(Y\right)$$

where ∇W(Y) is the Frechet derivative of the scalar differentiable function *W*, which is the strain energy density function of the elastic material. Please note that the deformation state *Y* considers material dependence on volume changes and shears. The following definitions (Equation (6–8)) of extension scalar state (*e),* influence function (*ω*) weighted volume (*m*) and scalar valued function dilatation (*θ*) are used towards that end.

(6) e=|Y|−|X|

(7) m=(ω|X|).|X|

(8) $$\theta =\frac{3}{m}\left(\omega \left|X\right|\right).e$$

where *e* signifies the change in bond length due to deformation; the influence function *ω* is a scalar state and *θ* is considered equal to the volumetric strain at small deformations obtained by the trace of linearized strain in classical theory. Thereafter, *e* can be decomposed into isotropic part (*e^i^* = *θ*|*X*|/3) and deviatoric part (*e^d^* = *e* − *e^i^*). Thus, *C* can be obtained from the partial derivatives of *W* with respect to *e^i^* and *e^d^* as shown in Equation (9).

(9) $$C=\frac{\partial W}{\partial \theta }\frac{\partial \theta }{\partial e^{i}}+\frac{\partial W}{\partial e^{d}}$$

For a linear peridynamic solid, the strain energy density function *W*(*θ*, *e^d^*) is defined as follows [1].

(10) $$W\left(\theta ,e^{d}\right)=\frac{K\theta^{2}}{2}+\frac{15G}{2m}\left(\omega e^{d}\right).e^{d}$$

where *K* and *G* are bulk and shear moduli of the material. Substituting *W*(*θ*, *e^d^*) (Equations (10)) in Equation (11), the force scalar state is obtained as shown in Equation (11).

(11) $$t=\frac{3K\theta }{m}\omega \left|X\right|+\frac{15G}{m}\omega e^{d}$$

Since the force scalar state determines the constitutive model shown in Equation (4), the choice of influence function *ω* can handle interfaces and free surfaces effectively. For a point *x* located near the boundary, the influence function is so chosen that it vanishes at all points in the horizon that lie outside the body. If the point *x* is near the interface of two different materials, two different influence functions can be chosen. The implementation of influence functions eliminates the surface effects observed in peridynamics [49]. The forthcoming subsection details the incorporation of damage in simulation framework.

#### 2.3.2. Damage Model

In peridynamics, material damage is introduced when interactions between material points are terminated. The existence of micro-potentials (interactions) is terminated between the material points when the bond between them is stretched beyond a threshold value. Such termination of interactions represents formation of a crack. In the peridynamic formulations, the breakage of a bond occurs independently among different bond lengths and orientations for a given particle. Thus, the initiation and growth process of cracks occurs without reference to any supplemental kinetic relation that controls crack growth. From this perspective, the fracture modeling is autonomous in such formulations in contrast to conventional methods [9]. To calculate the critical stretch that serves as the threshold for bond stretch beyond which damage initiates, the total work required to eliminate all interactions across the new surface is equated to the critical energy release rate. The critical stretch *s_c_* thus obtained is shown in Equation (12) [7].

(12) $$s_{c}=\sqrt{G_{c}/\left(\frac{6G}{\pi }+\frac{16\left(K-2G\right)}{9\pi^{2}}\right)\delta }$$

where *G_c_* is the critical energy release rate; *K* and *G* are the bulk and shear moduli respectively and *δ* is the horizon. The critical stretch is a function of the horizon *δ* which brings in the effect of physical material characteristics, nature of loading, length scale and computational cut-off radius. While *s_c_* serves as the damage initiation criterion, a history dependent scalar-valued function μ stores the damage states of such bonds. *µ* is defined as shown in Equation (13).

(13) $$\mu \left(x^{\prime }-x,t\right)=\{\begin{array}{c} \;1\;if\;bond\;sretch<s_{c}\\ 0\;otheriwse \end{array}$$

The function *µ* modifies the force state *T* to zero as the failure criterion is met which implies initiation of damage. Thus, the solution process involves iterative computation of displacements at each point followed by corresponding stretches between interacting points which terminates by assigning a zero value to *µ* as the stretch exceeds *s_c_*. The implementation of the history-dependent scalar function *µ* enables quantification of local damage at a point as shown in Equation (14) [13].

(14) $$\varphi \left(x,t\right)=1-\frac{\int_{H_{x}}^{}\mu \left(x^{\prime }-x,t\right)dV_{x’-x}}{\int_{H_{x}}^{}dV_{x’-x}}$$

where *φ*(*x*, *t*) defines local damage at point *x* as the weighted ratio of the number of eliminated interactions to the total number of initial interactions of the material point *x* with its family in *H_x_*. *φ* can range from 0 to 1 with 0 signifying that all the interactions are intact while 1 signifies termination of all such interactions. The local damage is an indicator of possible crack formation. Similar critical stretch-based failure criteria in peridynamic formulations has been successfully implemented and validated with experimental observations in [50]. The following sub-section elaborates the numerical implementation of the material and damage modules in a discretized framework.

In this paper, the peridynamic formulations are implemented in an open source program called Peridigm [13] developed at Sandia National Laboratories. The input file to Peridigm includes the discretization, block definition as described in Section 2.2; definition of material and damage models corresponding to blocks as described in Section 2.3.1 and 2.3.2 respectively followed by initialization of relevant boundary conditions at nodes and invoking the quasi-static solver. The solutions are passed on to the post-processor. Thereafter, it enables visualization in an open source visualization tool ParaView [51] and computes the engineering stresses and strains. The fundamental considerations for micromechanical analyses involving unit cells are dictated by the solution of the displacement field being its volume average in the unit cell, as shown in Equation (15).

(15) $$\bar{u}=\frac{1}{V}\overset{}{\underset{V}{\int }}udV$$

where ū is the displacement field in a higher scale with its representative unit cell embodying displacement u throughout the unit cell volume *V*. The displacement in the unit cell and its corresponding strain can be decomposed into averaged and fluctuation parts. The response in a macroscopically uniform deformation gradient can be obtained by unit cell response under equivalent loads. For uniaxial loading scenarios, the equivalent loads induce unit average strain thus enabling computation of the response of the unit cell which can thereafter characterize the constitutive response of the homogenized material at a higher scale. The uniqueness of the periodic conditions helps in simplification of the problems by ensuring equality between the fluctuating components on opposite faces.

## 3. Application of the Framework to Particulate Reinforced Cementitious Composites

**Figure 3:**
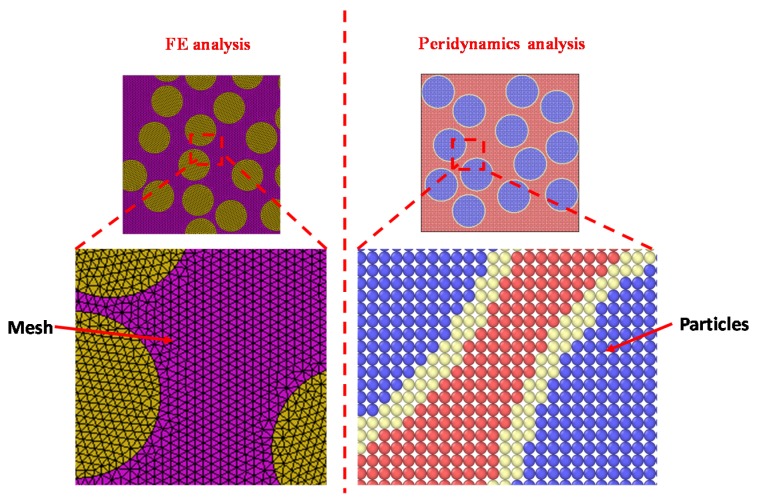
Sample mortar unit cell to describe the mesh-free approach in peridynamics as contrasted with the finite element (FE) mesh.

This section applies the aforementioned peridynamics-based framework towards performance-prediction of particulate-reinforced cementitious composite. Incorporation of waste iron powder replacing cement not only improves sustainability credentials of cementitious composite (due to reduction in cement-consumption) but also contributes towards enhanced mechanical behavior [38,39]. EAF (electric arc furnace) method of steel production and shot-blasting of structural steel sections generate a vast amount of the aforementioned waste iron powder which are landfilled at enormous environmental costs. Use of analytical homogenization techniques resulting in closed form solutions to predict constitutive relationships for such heterogenous systems is rendered ineffective owing to the stiffness contrasts in the component phases. A finite element (FE)-based study governed by cohesive laws for the interfaces and progressive damage in the matrix has been carried out for the metallic particulate-reinforced cementitious composite in the authors’ previous publication [39]. The damage law for the interface and the matrix in the FE study [39] are provided hereafter so as to provide insights into the merit of peridynamics application. The governing damage laws, used in the FE study, in the matrix are mentioned in Equation (16).

(16) $$D\left(\tilde{\varepsilon }\right)=1-\frac{\varepsilon_{D_{0}}\left(1-A_{t}\right)}{\tilde{\varepsilon }}-\frac{A_{t}}{\exp[B_{t}\left(\tilde{\varepsilon }-\varepsilon_{D_{0}}\right)]}$$

where *D* is the damage variable, $\tilde{\varepsilon }$ is the equivalent strain, $\varepsilon_{D_{0}}$ is the strain at damage initiation, *A_t_* and *B_t_* are material parameters. The traction-separation law governing the cohesive behavior in the interfaces is mentioned in Equation (17).

(17) $$\sigma_{c}=\{\begin{array}{c} K_{p}\lambda ,\;\;\lambda <\lambda_{0}\\ f_{t}exp^{\frac{-f_{t}\left(\lambda -\lambda_{0}\right)}{G_{F}}},\lambda \geq \lambda_{0} \end{array}$$

where σ_c_ is the traction, *λ* is the separation, K_p_ is the penalty stiffness, *G_F_* is the total fracture energy, *f_t_* is the tensile strength and *λ*_0_ is the threshold limit of separation. The formulations are adequately detailed in [39]. Herein, employing peridynamics leads to a non-local method well suited for modeling solid bodies with stiffness-mismatch. Unlike FE, spatial integral equations (sum of bond forces) are used in the peridynamic method which are defined even at discontinuities thereby reducing mathematical complexities. The failure in the current approach is governed by critical stretch-based method (See Equation (13)). Therefore, a multiscale numerical framework on iron powder modified mortars is undertaken towards prediction of effective constitutive response of such systems. Towards that end, peridynamics governed numerical homogenization is carried out at two distinct scales. Since explicit damage laws are not required in the peridynamics approach, the number of parameters essential for effective prediction of the material constitutive response decreases significantly. Additionally, the mesh-free approach enabled by particle generation in peridynamics is shown in Figure 3, as contrasted with the FE study [39] involving conventional meshes.

In both the interactive length-scales, the homogenization technique implements interfacial damage at the matrix-inclusion interface and enacts damage in the matrix thus capturing the composite constitutive behavior. A validation to the described multiscale numerical approach is realized by the strength and elastic parameters as compared with experimental observations. The simulated results are also compared with the FE results from the previous study [39] for more insights. The upcoming sub-sections detail the numerical simulation results enacted at multiple scales for effective property computations.

### 3.1. Effective Constitutive Response Prediction: A Multiscale Numerical Approach

The inherent heterogeneity of cementitious systems calls for an approach that can capture the complex microstructural features in randomly generated virtual microstructures while conserving the same across length scales of pastes and mortars. The numerical homogenization at the micro scale predicts the composite constitutive behavior of the waste iron powder modified cement paste. The homogenized constitutive behavior, thus obtained, serves as the matrix property for the meso-scale mortar model with sand inclusions. Thus, the current approach facilitates reproduction of microstructural information pertaining to various component phases as geometrical features in unit cells at distinct scales. The generated unit cells are thereafter discretized and subjected to suitable boundary conditions to simulate their effective constitutive response from a peridynamic perspective. The forthcoming sub-sections elaborate the unit cell generation, discretization, material and damage definitions and corresponding boundary conditions for each scale.

#### 3.1.1. Generation of Unit Cell and Discretization

**Figure 4:**
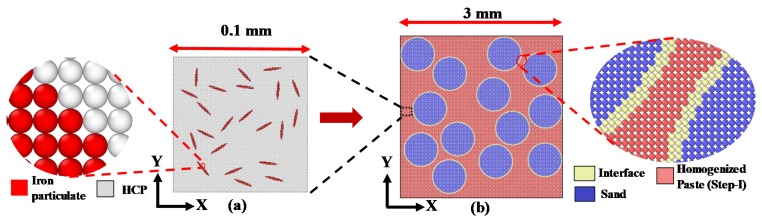
Interactive length scales: (**a**) Step I: 10% iron powder dispersed in hardened cement paste (HCP) matrix at micro-scale; (**b**) Step II: sand embedded in homogenized iron powder- HCP at meso-scale (the homogenized material from (**a**) serves as matrix for (**b**)).

The unit cells in both the length scales are obtained using the methodology described earlier in this paper (refer to Section 2.1 and Section 2.2). The generated unit cells for cement paste scale and mortar scale as shown in Figure 4a,b respectively for a characteristic mixture replacing 10% cement with iron powder. The constitutive response of the micro-scale model (Figure 4a) is extracted and assigned to the matrix of mesoscale model (Figure 4b). While the micro-scale geometry is characterized by waste iron powder inclusions in cement paste, the mesoscale geometry contains sand particles dispersed in the homogenized matrix (obtained from micro-scale). In the following sections, the generated micrographs and matrix/interface damage simulations are shown for the representative sample (10% iron powder replacing cement). The results from the microstructure guided numerical simulations include those of the control specimens and varying iron powder dosage. A comparative evaluation among the specimens has been presented thereafter. The median inclusion sizes adopted from [38,39] are 20 µm and 600 µm for iron particulates and sand inclusions, respectively. The respective aspect ratios are 12 (iron particulates) and 1(sand) [39,52]. The paste-sand interface is considered 20 µm thick [53,54,55,56]. The random locations and orientations of the inclusions in the periodic unit cells are obtained using the algorithm described earlier (See Section 2.1). The sizes of the unit cells are chosen to be 5 times the size of the inclusions which shows sufficient convergence [48]. The changes in constitutive response for sizes beyond the adopted unit cells are deemed insignificant.

The discretization technique discussed in Section 2.2 enables the distinction between various phases as shown in the zoomed pictures of Figure 4 for each scale which enables a block-based material definition as explained later. A judicious choice of the grid size and the resulting horizon results in a computationally efficient framework [57] with a stable solution. Here a grid size of 0.005 mm and 0.01 mm is chosen here for micro and meso-scale respectively. The horizons are taken to be 3.015 times the grid spacing to remove mathematical instabilities [48]. The grid spacing and horizon, adopted for each length scale, sufficiently represents the geometrical features and are found to yield convergence for the computed stress-strain curves. Although the framework presented in the paper can be effectively applied to 3D unit cells, a trade-off between computational demand and efficiency has been struck by analyzing 2D unit cells for the comparative evaluation. Such 2D unit cells have been successfully implemented in peridynamic formulations in [10].

#### 3.1.2. Blocks: Material and Damage Definition

The input code in Peridigm assigns material properties to each material block in the discretized domain corresponding to every phase of the microstructure. The material input properties required for formulations in Section 2.3.1 and Section 2.3.2 are the bulk and shear moduli for defining the linear peridynamic solid and the critical energy release rate to initialize the damage criterion. The input Young’s modulus for the cement paste matrix, sand and iron particulates are 20, 70 and 200 GPa respectively [29,58]. A constant Poisson’s ratio of 0.2 is considered for all the materials except the iron particles since a range of 0.17–0.22 for the same yields insignificant changes in the results [59,60]. A Poisson’s ratio of 0.3 is adopted for iron particulates [39,61]. Owing to lack of data. the matrix properties are assigned to the iron particulate-HCP interface elements as well. Similar properties have been successfully adopted in [59,61]. The bulk and shear moduli are thus computed from the Young’s modulus and the Poisson’s ratio for each phase of the micro-scale unit cell. The HCP matrix implemented in the micro-scale simulations have a critical energy release rate of 0.017 N/mm [39]. The constitutive response of the iron powder modified cement pastes (output from the micro-scale post-processor) characterizes the matrix properties of the meso-scale mortar model. The sand-matrix interface is considered to have elastic properties a third of the surrounding matrix [62]. To characterize the damage in the meso-scale, the following formulations are used to obtain the *G_c_* [31].

(18) $$\left(1-D\right)\varepsilon =\varepsilon_{D_{0}}\exp\left(-\frac{Dh\varepsilon f_{t}}{G_{C}}\right)$$

where *D* is damage variable (0 < *D* < 1) relating stress tensor *σ* with strain *ε* in terms of elasticity tensor *E* as σ = (1 − D)E:ε; ε is the tensile strain reaching $\varepsilon_{D_{0}}$ when tensile strength reaches *f_t_*; h is the centroidal mean distance of adjacent elements (here, grid spacing) and *G_C_* is strain energy release rate. Here, the value of *D* is considered 0.9 and the corresponding strain *ε* is adopted. The values of $\varepsilon_{D_{0}}$ and *f_t_* are obtained from the effective constitutive relation of the material. This enables definition of *s_c_* as per Equation (12). The identified material parameters for the micro-scale iron-powder modified cement pastes are presented in Table 1 for different dosages of waste iron powder. These parameters serve as input for the meso-scale simulations.

#### 3.1.3. Effective Constitutive Responses at Multiple Scales

**Figure 5:**
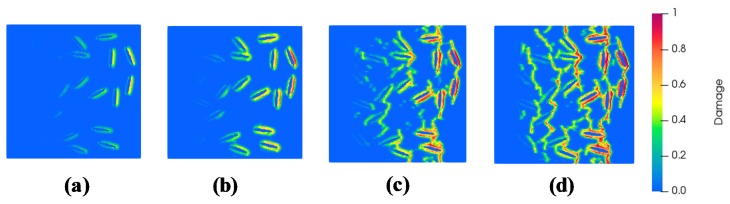
Progressive damage observed in iron powder (10%) modified HCP corresponding to applied tensile strains of (**a**) 24 *µε*, (**b**) 78 *µε*, (**c**) 121 *µε* and (**d**) 130 *µε*.

The results from the numerical homogenization carried out at multiple scales are reported in the current section. The procedure initiates with a microstructure guided numerical homogenization at the micro-scale (See Figure 4a). A uniaxial tensile strain along X is applied to the discretized unit cell. This is implemented in the input file to Peridigm as a nodal displacement boundary condition that ensures a constrained left edge along X. The nodes are subjected to a velocity simulating a quasi-static strain rate. The material properties as reported in Section 3.1.2 for the hardened cement paste (HCP) without any iron content are adopted. It is to be noted that the micro-scale matrix is HCP with a water-cement ratio of 0.5 by mass for all the digital specimens with varying iron powder contents. The analysis is carried out using the quasi-static solver and the simulation yields progressive damage as shown in Figure 5.

**Figure 6:**
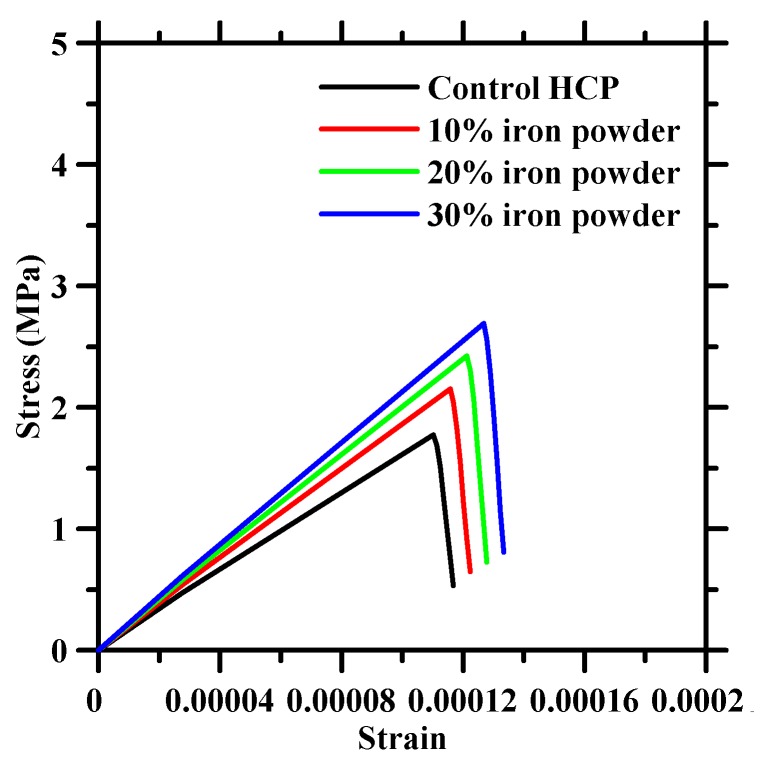
Simulated tensile constitutive response of pastes with varying iron powder dosage in HCP.

Figure 5 shows the progressive damage when the digital specimens are subjected to uniaxial strains of 24, 78, 21, and 130 *µε*. The damage initiates at the iron particulate-HCP interfaces and propagates with increasing strains. Beyond the peak strain, the damages along the interfaces coalesce thus initiating matrix damage. Thereafter, matrix damage continues to propagate with increasing strains. Figure 6 shows the constitutive response for tensile loading of iron powder modified cement paste for varying iron powder dosages clearly illustrating the gain in tensile strength with higher dosages.

**Figure 7:**
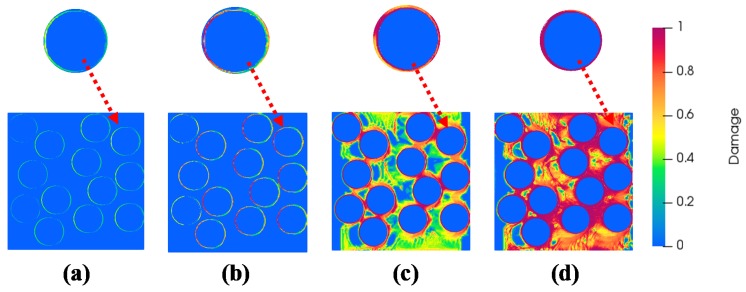
Progressive damage (interface/matrix) observed in iron powder (10%) modified mortar corresponding to applied tensile strains of (**a**) 52 *µε*, (**b**) 105 *µε,* (**c**) 153 *µε* and (**d**) 208 *µε* (the interface damage is highlighted in zoomed pictures).

The extracted tensile constitutive response of the pastes are thereafter applied as matrix properties in the mortars (see Figure 4b). A similar procedure of assigning material properties to the blocks followed by application of tensile strain is followed. The progressive interface and matrix damage for iron particulate (10%) modified mortars are presented in Figure 7.

**Figure 8:**
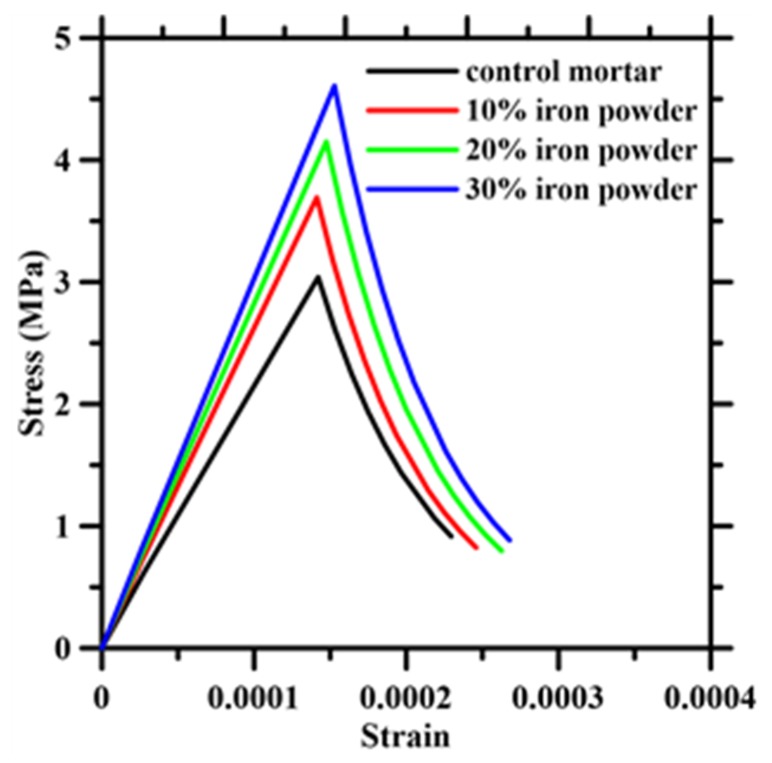
Simulated tensile constitutive response of mortars with varying iron powder dosage.

Figure 7 presents the interface damage and matrix damage under applied strain of 52 *µε*, 105 *µε*, 153 *µε* and 208 *µε*. A weaker interface implies onset of damage much lower than the peak strain. As the interfacial damage propagates, the stress in the matrix keeps on increasing. The debonding brought about the interfacial damage terminates at the point of initiation of matrix damage which corresponds to the matrix tensile strength. For higher strains, the damage propagates in the matrix thereby characterizing the post-peak response. The tensile constitutive behavior of the simulated mortars are shown in Figure 8 with varying iron powder dosages.

The current framework effectively captures the heterogeneities in particulate modified cement pastes and predicts their constitutive responses with varying iron powder contents. The three-phase interactions involving stiff inclusions in a weak matrix surrounded by a weaker interface are enabled by peridynamic formulations with critical stretch-based failure. Progressive failure is captured in the interface and the matrix leading to accumulated damage in the representative unit cells.

### 3.2. Comparison with Experimental Observations

**Figure 9:**
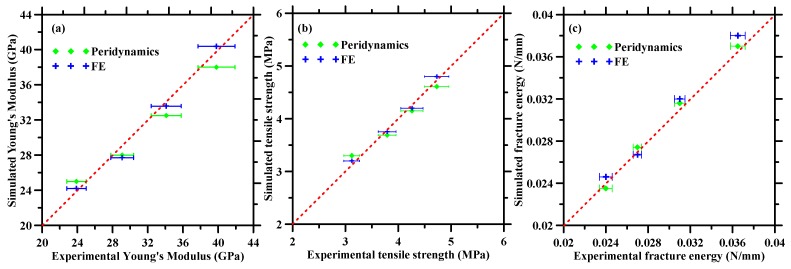
Correlation between experimental and simulated (peridynamics and finite element (FE) approach) (**a**) Young’s modulus and (**b**) tensile strengths and (**c**) fracture energy of mortars with various dosage of waste iron powder.

This section draws a comparison between the simulated responses and the experimental observations [39]. In addition, the FE-based simulation results, obtained from a previous publication [39], are also plotted for a comparative evaluation. Figure 9 reports the comparison of Young’s modulus, tensile strength, and fracture energy. Equation (18) enables calculation of fracture energy from the effective constitutive response as shown in Figure 8. The simulated responses from peridynamics simulations show excellent match with the experimental observations as well as FE results for various dosage of iron powder. The results suggest that both FE and peridynamics-based approaches can adequately predict the mechanical responses in metallic particulate-reinforced cementitious composites. However, the peridynamics-based approach requires fewer input parameters as compared to FE-based approach which signifies the efficacy of the peridynamics-based multiscale numerical simulation approach presented in this paper.

## 4. Conclusions

The study elaborates a peridynamics-based micromechanical simulation framework for random heterogenous composites. The conclusions are mentioned herewith.

The microstructural features of the composite are effectively captured into the framework by means of representative unit cells with multiple phases that are discretized into distinct blocks thus enabling material property application.

- The peridynamic formulations allows spontaneous damage initiation and propagation based on critical stretch criterion.
- The framework effectively integrates the phase separated microstructure in a peridynamic solver that applies a uniaxial strain to characterize the composite constitutive response for tensile loading. The framework is thereafter applied for random heterogenous microstructures of metallic particulate reinforced cement-based composites in a multiscale approach with a view to assess the capability of the numerical framework.
- The multiple length scales involve microscale simulations for iron inclusions embedded in cement paste, the properties of which are homogenized to form the input to the matrix of the meso-scale mortar with sand inclusions. Thereafter, the framework is applied in the meso-scale to obtain simulated effective constitutive responses of the mortars.
- The simulated Young’s modulus, tensile strength and fracture energy for iron powder-incorporated cementitious composites are compared with experimental observations which shows a close correlation thereby validating the framework.
- The comparison between the simulation results obtained from FE analysis and peridynamics approach establishes the efficiency of the peridynamics approach in capturing material responses with fewer input parameters.

Overall, the peridynamics-based approach can handle discontinuities arising out of deformities in a robust and efficient computation that involves integral equations unlike the differential counterparts used in classical continuum mechanics. Additionally, the implementation of state-based peridynamics provides extensibility to conventional constitutive material models and nano-scale molecular dynamic-based simulations alike. The predictive tool thus developed potentially provides an efficient means to customize the microstructure of a variety of inclusion-incorporated composites for optimized performance.

## Figures and Tables

**Table 1:** Parameters and material properties for iron powder incorporated HCP.

	% Iron Powder	E (GPa)	f_t_ (MPa)	Peak Strain	G_C_ (N/mm)
Micro scale	0	20	1.77	0.000111	0.017
	10	22.25279	2.15	0.000118	0.02
	20	24.98885	2.42	0.000125	0.023
	30	25.66295	2.69	0.000131	0.025

## References

[1] Silling S.A., Epton M., Weckner O., Xu J., Askari E. (2007). Peridynamic States and Constitutive Modeling. J. Elast..

[2] Silling S.A., Askari E. (2005). A meshfree method based on the peridynamic model of solid mechanics. Comput. Struct..

[3] Macek R.W., Silling S.A. (2007). Peridynamics via finite element analysis. Finite Elem. Anal. Des..

[4] Silling S.A., Zimmermann M., Abeyaratne R. (2003). Deformation of a Peridynamic Bar. J. Elast..

[5] Silling S.A. (2000). Reformulation of elasticity theory for discontinuities and long-range forces. J. Mech. Phys. Solids.

[6] Foster J.T., Silling S.A., Chen W.W. (2010). Viscoplasticity using peridynamics. Int. J. Numer. Methods Eng..

[7] Madenci E., Oterkus E., Madenci E., Oterkus E. (2014). Peridynamic Theory. Peridynamic Theory and Its Applications.

[8] Silling S.A., Askari E. (2004). Peridynamic Modeling of Impact Damage.

[9] Silling S.A., Bobaru F. (2005). Peridynamic modeling of membranes and fibers. Int. J. Nonlinear Mech..

[10] Vogler T., Lammi C.J. (2014). A Nonlocal Peridynamic Plasticity Model for the Dynamic Flow and Fracture of Concrete.

[11] Emmrich E., Lehoucq R.B., Puhst D., Griebel M., Schweitzer M.A. (2013). Peridynamics: A Nonlocal Continuum Theory. Meshfree Methods for Partial Differential Equations VI.

[12] Breitenfeld M.S., Geubelle P.H., Weckner O., Silling S.A. (2014). Non-ordinary state-based peridynamic analysis of stationary crack problems. Comput. Methods Appl. Mech. Eng..

[13] Parks M.L., Littlewood D.J., Mitchell J.A., Silling S.A. (2012). Peridigm Users’ Guide V1.0.0.

[14] Le Q.V., Chan W.K., Schwartz J. (2014). A two-dimensional ordinary, state-based peridynamic model for linearly elastic solids. Int. J. Numer. Methods Eng..

[15] Askari E., Bobaru F., Lehoucq R.B., Parks M.L., Silling S.A., Weckner O. (2008). Peridynamics for multiscale materials modeling. J. Phys. Conf. Ser..

[16] Colavito K., Kilic B., Celik E., Madenci E., Askari E., Silling S. Effect of Void Content on Stiffness and Strength of Composites by Peridynamic Analysis and Static Indentation Test. Proceedings of the 48th AIAA/ASME/ASCE/AHS/ASC Structures, Structural Dynamics, and Materials Conference.

[17] Hu Y.L., Madenci E. (2017). Peridynamics for fatigue life and residual strength prediction of composite laminates. Compos. Struct..

[18] Ghajari M., Iannucci L., Curtis P. (2014). A peridynamic material model for the analysis of dynamic crack propagation in orthotropic media. Comput. Methods Appl. Mech. Eng..

[19] De Meo D., Zhu N., Oterkus E. (2016). Peridynamic Modeling of Granular Fracture in Polycrystalline Materials. J. Eng. Mater. Technol..

[20] Huang D., Zhang Q., Qiao P. (2011). Damage and progressive failure of concrete structures using non-local peridynamic modeling. Sci. China Technol. Sci..

[21] Mori T., Tanaka K. (1973). Average stress in matrix and average elastic energy of materials with misfitting inclusions. Acta Metall..

[22] Hori M., Nemat-Nasser S. (1993). Double-inclusion model and overall moduli of multi-phase composites. Mech. Mater..

[23] Yang C.C., Huang R. (1996). Double inclusion model for approximate elastic moduli of concrete material. Cem. Concr. Res..

[24] Das S., Aguayo M., Kabay N., Mobasher B., Sant G., Neithalath N. (2018). Elucidating the influences of compliant microscale inclusions on the fracture behavior of cementitious composites. Cem. Concr. Compos..

[25] Das S., Aguayo M., Rajan S.D., Sant G., Neithalath N. (2018). Microstructure-guided numerical simulations to predict the thermal performance of a hierarchical cement-based composite material. Cem. Concr. Compos..

[26] Aguayo M., Das S., Maroli A., Kabay N., Mertens J.C.E., Rajan S.D., Sant G., Chawla N., Neithalath N. (2016). The influence of microencapsulated phase change material (PCM) characteristics on the microstructure and strength of cementitious composites: Experiments and finite element simulations. Cem. Concr. Compos..

[27] Chawla N., Chawla K.K. (2006). Microstructure-based modeling of the deformation behavior of particle reinforced metal matrix composites. J. Mater. Sci..

[28] Padilla E., Jakkali V., Jiang L., Chawla N. (2012). Quantifying the effect of porosity on the evolution of deformation and damage in Sn-based solder joints by X-ray microtomography and microstructure-based finite element modeling. Acta Mater..

[29] Das S., Maroli A., Singh S.S., Stannard T., Xiao X., Chawla N., Neithalath N. (2016). A microstructure-guided constitutive modeling approach for random heterogeneous materials: Application to structural binders. Comput. Mater. Sci..

[30] Grassl P., Grégoire D., Rojas Solano L., Pijaudier-Cabot G. (2012). Meso-scale modelling of the size effect on the fracture process zone of concrete. Int. J. Solids Struct..

[31] Grassl P., Jirásek M. (2010). Meso-scale approach to modelling the fracture process zone of concrete subjected to uniaxial tension. Int. J. Solids Struct..

[32] van Mier J.G.M. (2017). Fracture Processes of Concrete.

[33] Wells G.N., Sluys L.J. (2001). A new method for modelling cohesive cracks using finite elements. Int. J. Numer. Methods Eng..

[34] Chen L., Rabczuk T., Bordas S.P.A., Liu G.R., Zeng K.Y., Kerfriden P. (2012). Extended finite element method with edge-based strain smoothing (ESm-XFEM) for linear elastic crack growth. Comput. Methods Appl. Mech. Eng..

[35] de Borst R. (2003). Numerical aspects of cohesive-zone models. Eng. Fract. Mech..

[36] Papoulia K.D., Sam C.-H., Vavasis S.A. (2003). Time continuity in cohesive finite element modeling. Int. J. Numer. Methods Eng..

[37] Zi G., Rabczuk T., Wall W. (2007). Extended meshfree methods without branch enrichment for cohesive cracks. Comput. Mech..

[38] Das S., Kizilkanat A., Neithalath N. (2015). Crack propagation and strain localization in metallic particulate-reinforced cementitious mortars. Mater. Des..

[39] Nayak S., Krishnan N.M.A., Das S. (2019). Fracture response of metallic particulate-reinforced cementitious composites: Insights from experiments and multiscale numerical simulations. Cem. Concr. Compos..

[40] Lubachevsky B.D., Stillinger F.H., Pinson E.N. (1991). Disks vs. spheres: Contrasting properties of random packings. J. Stat. Phys..

[41] Lubachevsky B.D., Stillinger F.H. (1990). Geometric properties of random disk packings. J. Stat. Phys..

[42] Meier H.A., Kuhl E., Steinmann P. (2008). A note on the generation of periodic granular microstructures based on grain size distributions. Int. J. Numer. Anal. Methods Geomech..

[43] Das S., Maroli A., Neithalath N. (2016). Micromechanical Modeling for Material Design of Durable Infrastructural Materials: The Influence of Aggregate and Matrix Modification on Elastic Behavior of Mortars. Int. Conf. Durab. Concr. Struct..

[44] van der Sluis O., Schreurs P.J.G., Brekelmans W.A.M., Meijer H.E.H. (2000). Overall behaviour of heterogeneous elastoviscoplastic materials: Effect of microstructural modelling. Mech. Mater..

[45] Raghavan P., Ghosh S. (2005). A continuum damage mechanics model for unidirectional composites undergoing interfacial debonding. Mech. Mater..

[46] Mohsen K., Straatman A.G. (2007). A thermal periodic boundary condition for heating and cooling processes. Int. J. Heat Fluid Flow.

[47] Sanahuja J., Toulemonde C. (2011). Numerical homogenization of concrete microstructures without explicit meshes. Cem. Concr. Res..

[48] Tang L., Krishnan N.M.A., Berjikian J., Rivera J., Smedskjaer M.M., Mauro J.C., Zhou W., Bauchy M. (2018). Effect of nanoscale phase separation on the fracture behavior of glasses: Toward tough, yet transparent glasses. Phys. Rev. Mater..

[49] Le Q.V., Bobaru F. (2018). Surface corrections for peridynamic models in elasticity and fracture. Comput. Mech..

[50] Ayatollahi M.R., Aliha M.R.M. (2009). Analysis of a new specimen for mixed mode fracture tests on brittle materials. Eng. Fract. Mech..

[51] Ahrens J., Geveci B., Law C. (2005). ParaView: An End-User Tool for Large-Data Visualization. Visualization Handbook.

[52] Das S., Maroli A., Neithalath N. (2016). Finite element-based micromechanical modeling of the influence of phase properties on the elastic response of cementitious mortars. Constr. Build. Mater..

[53] Scrivener K.L., Crumbie A.K., Laugesen P. (2004). The Interfacial Transition Zone (ITZ) Between Cement Paste and Aggregate in Concrete. Interface Sci..

[54] Ollivier J.P., Maso J.C., Bourdette B. (1995). Interfacial transition zone in concrete. Adv. Cem. Based Mater..

[55] Bentz D.P. (2009). Influence of internal curing using lightweight aggregates on interfacial transition zone percolation and chloride ingress in mortars. Cem. Concr. Compos..

[56] Grondin F., Matallah M. (2014). How to consider the Interfacial Transition Zones in the finite element modelling of concrete?. Cem. Concr. Res..

[57] Ren H., Zhuang X., Rabczuk T. (2017). Dual-horizon peridynamics: A stable solution to varying horizons. Comput. Methods Appl. Mech. Eng..

[58] Nayak S., Das S. (2019). A microstructure-guided numerical approach to evaluate strain sensing and damage detection ability of random heterogeneous self-sensing structural materials. Comput. Mater. Sci..

[59] Das S., Yang P., Singh S.S., Mertens J.C.E., Xiao X., Chawla N., Neithalath N. (2015). Effective properties of a fly ash geopolymer: Synergistic application of X-ray synchrotron tomography, nanoindentation, and homogenization models. Cem. Concr. Res..

[60] Da S., Němeček J., Štemberk P. (2013). Application of multiscale elastic homogenization based on nanoindentation for high performance concrete. Adv. Eng. Softw..

[61] Yang P., Chowdhury S., Neithalath N. (2018). Strain sensing ability of metallic particulate reinforced cementitious composites: Experiments and microstructure-guided finite element modeling. Cem. Concr. Compos..

[62] Tijssens M.G.A., Sluys L.J., van der Giessen E. (2001). Simulation of fracture of cementitious composites with explicit modeling of microstructural features. Eng. Fract. Mech..

